# Coping strategies as predictors of pain and disability in older people in primary care: a longitudinal study

**DOI:** 10.1186/1471-2296-14-67

**Published:** 2013-05-24

**Authors:** Kay Benyon, Sara Muller, Susan Hill, Christian Mallen

**Affiliations:** 1 Primary Care Sciences, Keele University, Staffordshire, ST5 5BG, UK

**Keywords:** Coping, Catastrophizing, Primary care, Musculoskeletal pain, Longitudinal study

## Abstract

**Background:**

Musculoskeletal pain in older adults is common. It is hypothesised that coping strategies may be predictive of pain intensity and pain-related disability at six months after initial consultation in primary care.

**Method:**

Consecutive patients aged fifty years and over with musculoskeletal pain were recruited from general practice consultations. A self-completion postal questionnaire was sent to participants at baseline, with a follow-up questionnaire mailed six months later. Coping was assessed using The Coping Strategies Questionnaire (CSQ), pain and pain related disability were measured using domains of The Chronic Pain Grade (CPG). Associations between coping strategies and pain and disability were investigated using ordinary least squares regression.

**Results:**

Crude analysis revealed catastrophizing at baseline was predictive of higher levels of pain and disability at baseline and was predictive of disability at six months. The association between catastrophizing and pain and pain related disability at follow-up was not significant once adjustments were made for age, gender and baseline anxiety and depression. Increasing behaviour and self-statements were not associated with pain or disability at follow-up. Ignoring pain sensations was predictive of increased pain at follow-up.

**Conclusion:**

This study highlights the relationship between catastrophizing in predicting pain and pain related disability may be mediated by other factors such as anxiety and depression. Ignoring sensations in those with high levels of pain may be maladaptive in older people with musculoskeletal pain.

## Background

Musculoskeletal pain is common, representing more than one in seven primary care consultations [1]. Recently, there has been an increased focus on factors that may be associated with the prognosis of musculoskeletal pain, with particular interest in the significance of coping strategies and the scope for modification to improve outcome [2,3].

Coping as defined by Lazarus and Folkman [4] is ‘a process of evaluating a stressful situation consisting of primary and secondary appraisal and responding to a stressor’. Coping with medical conditions may also be influenced by the illness perceptions of an individual and the emotional response to the perceived health threat [5]. The coping strategies people inherently use may have potential to affect the outcome of their condition. There is an abundance of cross-sectional studies [6-10] which have demonstrated an association between coping strategies and pain and disability, particularly for patients with low back pain. Catastrophizing (defined as concentrating on the magnitude of a stressful situation [11]) has been shown to be associated with negative outcome in several cross-sectional studies [6,7,10]. Passive coping strategies such as surrendering the control of the situation have also been shown to correlate with increased pain and disability in adults with osteoarthritis [8,9]. Whilst these studies are valuable in demonstrating a potential relationship they are unable to confirm whether individual coping strategies are predictive of pain over time in primary care.

Systematic reviews [12-15] of longitudinal studies have investigated whether coping strategies are predictive of long term outcomes. These reviews have demonstrated how some coping strategies such as catastrophizing and passive coping correlate with a poor outcome in patients with back pain. However, for other musculoskeletal pain disorders, including osteoarthritis, reviews have concluded there is insufficient research in this area to form firm conclusions. The aim of this paper is to investigate whether coping strategies are associated with pain and pain related disability within primary care in adults aged fifty years and over with musculoskeletal pain over a six-month period. It is hypothesized that baseline pain and disability influence coping and consequently pain and disability at follow-up. A secondary objective of this study is to compare the associations of coping and outcome in those with high and low baseline pain and disability.

## Methods

### Study design and population

The PROGnostic RESearch (PROG-RES) study is a prospective observational cohort study conducted in six general practices in Cheshire, United Kingdom. Recruitment took place between September 2006 and March 2007. During the consultation an electronically activated template was triggered when a musculoskeletal Read code was entered onto the Egton Medical Information System (EMIS). This computer system is used by around 50% of primary care physicians in the UK. Patients were eligible for the study if they met the following inclusion criteria: aged fifty years and over, consulting with musculoskeletal pain, registered with one of the participating practices during the study time and able to provide written informed consent. Exclusion criteria included patients with inflammatory arthropathies, vulnerable groups such as those unable to give consent, patients exhibiting signs and symptoms of underlying serious pathology such as an acute, red, hot, swollen joint or recent trauma associated with significant injury. The exclusion criteria were applied within the consultation, at the discretion of the general practitioner. The patient’s electronic medical record was tagged so they could not be sampled more than once and they were then contacted by the research team via mail. Weekly electronic searches were performed to identify tagged records in the preceding week and follow-up occurred 6 months after the index consultation.

Eligible patients were mailed a package consisting of baseline study questionnaire, consent form, and information on study participation. If there was no response at initial study invitation, a reminder postcard was mailed two weeks later and a further questionnaire in the following two weeks. Those participants who consented and completed the baseline measures, received a questionnaire package six months later. The baseline questionnaire provided questions on demographics, coping strategies used, musculoskeletal pain and disability in the preceding three months, other information such as if the person had emotional support, symptoms of depression and anxiety were also included. The six month follow-up questionnaire assessed pain and disability in the previous three months. For further details, the full methodology is described elsewhere [16]. Ethical approval was received from the Central Cheshire Local Research Ethics Committee.

### Measures

Pain and disability were measured using the Chronic Pain Grade (CPG) [17]. The CPG is a seven item instrument that provides a 0–100 scale for each domain of pain and disability. A recent systematic review has demonstrated this instrument to have good psychometric properties, including internal consistency, reliability and validity [18]. The pain and disability domains can be combined with a further item regarding limitation in usual activities, to give a grade from 0 to IV. However for the purpose of this study, the pain and disability domain scores were considered separately.

An amended version of the Coping Strategies Questionnaire (CSQ) [19], using only two items to assess each domain, was used at baseline to assess four coping strategies: catastrophizing, ignoring pain sensations, increasing behaviour and using coping self-statements. The psychometric properties of the long form of the CSQ have been extensively tested, and the shorter version used in the current study has been shown to provide approximations to the longer questionnaire [19]. The CSQ items used can be viewed in Appendix 1. Each item was scored from zero to six, with zero representing ‘no use’ and six representing ‘regular use’ of the coping strategy. Scores for each domain are the mean score of the two items in that domain.

### Statistical analysis

First, associations of baseline pain with baseline coping strategies were estimated using ordinary least squares regression models. These crude estimates were then adjusted for age, gender and baseline anxiety and depression (Table 1). These analyses were repeated with baseline disability as the outcome, again adjusting the model for age, gender and baseline anxiety and depression.

**Table 1:** Association between coping strategies and baseline pain and baseline disability

	**Regression coefficient (95%)**			
	**Pain**		**Disability**	
	**Unadjusted**	**Adjusted **^**ψ**^	**Unadjusted**	**Adjusted **^**ψ**^
Catatrophizing	5.93 (5.13, 6.72)	5.15 (4.25, 6.05)	8.44 (7.23, 9.65)	6.92 (5.54, 8.31)
Ignoring	−0.85 (−2.00, 0.31)	−0.10 (−1.18, 0.98)	−2.74 (−4.48, –1.00)	−1.72 (−3.36, –0.08)
Self-statements	−0.03 (−1.19, 1.13)	0.67 (−0.40, 1.74)	−1.48 (−3.25, 0.28)	−0.32 (−1.93, 1.30)
Increasing behaviour	0.20 (−0.99, 1.39)	0.93 (−1.17, 2.02)	−0.78 (−2.59, 1.02)	0.38 (−1.28, 2.04)

^ψ^ Age, gender, anxiety and depression adjusted.

Second, the association between baseline coping strategies and pain and disability at follow up were estimated. This analysis was adjusted for age, gender and baseline pain, disability, anxiety and depression (Table 2). In order to establish whether the association between coping strategies and pain at follow-up was similar in people with baseline pain above and below the median, regression analyses were repeated in these two pain groups. Similarly, the analysis of the association between baseline coping strategies and disability at follow-up were stratified according to baseline disability level (Table 3). All results are presented as regression coefficients with confidence intervals (CI) of 95%. All analyses were conducted in Stata 12.1 [20].

**Table 2:** Association between coping strategies and 6 month outcomes

	**Regression coefficient (95% CI)**			
	**Pain**		**Disability**	
	**Unadjusted**	**Adjusted**^**‡**^	**Unadjusted**	**Adjusted**^**‡**^
Catastrophizing	5.58 (4.09, 7.06)	1.09 (−0.81, 2.98)	6.84 (5.30, 8.38)	1.76 (−0.04, 3.56)
Ignoring	1.68 (−0.25, 3.61)	2.09 (0.31, 3.87)	0.72 (−1.34, 2.78)	1.70 (0.03, 3.37)
Self-statements	0.46 (−1.53, 2.44)	0.94 (−0.87, 2.74)	−0.51 (−2.65, 1.62)	0.27 (−1.44, 1.99)
Increasing behaviour	0.19 (−1.81, 2.19)	0.24 (−1.58, 2.07)	−0.51 (−2.67, 1.65)	−0.51 (−2.26, 1.23)

^‡^ Age, gender, BL pain, disability, anxiety and depression adjusted.

**Table 3:** Association between coping strategies and 6 month pain and disability: by baseline pain and disability groups

	**Regression coefficient (95% CI)**							
	**Outcome: 6 month pain score**				**Outcome: 6 month disability score**			
	**Baseline pain below median**		**Baseline pain above median**		**Baseline disability below median**		**Baseline disability above median**	
	**Unadjusted**	**Adjusted **^**ψ**^	**Unadjusted**	**Adjusted **^**ψ**^	**Unadjusted**	**Adjusted**^**‡**^	**Unadjusted**	**Adjusted**^**‡**^
Catastrophizing	3.57 (1.21, 5.94)	2.57 (−0.01, 5.14)	2.95 (0.63, 5.26)	0.86 (−1.96, 3.68)	4.17 (2.16, 6.18)	1.62 (−0.49, 3.73)	4.92 (2.44, 7.40)	2.73 (−0.27, 5.74)
Ignoring	1.22 (−1.47, 3.92)	0.75 (−1.93, 3.42)	2.50 (0.14, 4.86)	3.36 (0.92, 5.80)	0.15 (−2.27, 2.56)	−0.48 (−2.56, 1.59)	1.13 (−1.63, 3.90)	2.51 (−0.18, 5.21)
Self-statements	1.20 (−1.22, 3.63)	1.41 (−0.92, 3.74)	0.78 (−1.97, 3.52)	1.631 (−1.57, 4.20)	−0.01 (−2.45, 2.23)	0.16 (−1.72, 2.04)	−1.02 (−4.20, 2.15)	0.06 (−3.11, 3.22)
Increasing behaviour	0.73 (−1.70, 3.17)	0.83 (−1.49, 3.15)	−0.32 (−3.07, 2.42)	0.79 (−2.13, 3.71)	1.02 (−1.20, 3.24)	0.28 (−1.60, 2.17)	−3.02 (−6.33, 0.29)	−2.34 (−5.67, 0.99)

^ψ^ Age, gender, BL disability, anxiety and depression adjusted.

^‡^ Age, gender, BL pain, anxiety and depression adjusted.

## Results

At baseline, 650 questionnaires were mailed and 502 (77.2%) people responded. Participants had a mean age of 65.2 years (SD 10.2) and 306 (61.2%) were female. Details of the baseline characteristics of the study group are summarized in Table 4. At baseline, the mean pain score was 65.69 (SD 19.2) and the mean disability score was 50.53 (SD 28.9). In comparing the group who had below median pain and those who had above median pain, the group with the higher pain level was made up of a higher proportion of females, and a higher proportion of people with anxiety, depression and worse general health. Those who were in the above median disability groups tended to be more likely to have depression and anxiety, reported a worse general health and catastrophized more (data not shown). Of the 502 people who responded at baseline, 436 participants consented to follow-up. Therefore, at six months, 436 questionnaires were mailed and 370 (73.7% of the original cohort of 502) participants responded. There were no significant differences between the baseline characteristics of those who did and did not respond at follow-up.

**Table 4:** Demographics of participants split for above and below baseline median pain

**Demographics**	**Low baseline pain**	**High baseline pain**
Age		
50–59	96 (40.0)	73 (33.6)
60–69	86 (35.8)	69 (31.8)
70+	58 (24.2)	75 (34.6)
Gender		
Female	134 (56.1)	172 (65.9)
Male	105 (43.9)	89 (34.1)
HAD score Depression		
None (0–7)	208 (87.4)	124 (57.9)
Possible (8–11)	28 (11.8)	65 (30.4)
Probable (12–21)	2 (0.8)	25 (11.7)
HAD score Anxiety		
None (0–7)	161 (67.4)	96 (45.5)
Possible (8–11)	62 (25.9)	69 (32.7)
Probable (12–21)	16 (6.7)	46 (21.8)
Mean (SD) CSQ ignoring	2.97 (1.44)	2.71 (1.63)
Mean (SD) CSQ catastrophizing	1.67 (1.52)	3.42 (1.70)
Mean (SD) CSQ self statements	3.65 (1.60)	3.46 (1.44)
Mean (SD) CSQ increasing behaviour	3.75 (1.54)	3.73 (1.44)
Mean (SD) Baseline Pain score	50.42 (13.18)	80.70 (7.44)
Mean (SD) Baseline Disability score	34.63 (25.16)	66.18 (22.35)
Live alone?		
Yes	47 (19.6)	34 (15.8)
No	193 (80.4)	181 (84.2)
Emotional support?		
Yes	209 (87.1)	188 (88.7)
No	6 (2.5)	11 (5.2)
No need	25 (10.4)	13 (6.1)
Is there anyone to give you extra help?		
Yes	177 (73.8)	178 (83.2)
No	12 (5.0)	17 (7.9)
No need	51 (21.3)	19 (8.9)
Employment status		
Employed	86 (36.8)	53 (25.4)
Ill health	9 (3.9)	20 (9.6)
Retired	113 (48.3)	112 (53.6)
Unemployed	2 (0.9)	0 (0.0)
Housewife	18 (7.7)	19 (9.1)
Other	6 (2.6)	5 (2.4)
General health		
Excellent	16 (6.8)	6 (2.8)
Very good	77 (32.6)	34 (16.0)
Good	96 (40.7)	88 (41.5)
Fair	43 (18.2)	66 (31.1)
Poor	4 (1.7)	18 (8.5)

Full details of unadjusted and adjusted regression coefficients at baseline are shown in Table 1. At baseline catastrophizing was shown to be associated with more pain (regression coefficient (β) (increase in pain score for 1 point increase in catastrophising) 5.93, 95% CI 5.13, 6.72). Once this was adjusted for age, gender, anxiety and depression, the association between catastrophizing and pain was attenuated, but still significant (β =5.15 95% CI 4.25, 6.05). Catastrophizing was also positively associated with disability both in the unadjusted (β = 8.44, 95% CI 7.23, 9.65) and adjusted models (β = 6.92, 95% CI 5.54, 8.31). Ignoring sensations was association with a decrease in baseline disability (adjusted β –1.72, 95% CI −3.36, –0.08), but not with pain. Self-statements and increasing behaviours were not statistically associated with baseline pain or disability.

Details of the unadjusted and adjusted regression coefficients at six months for pain and disability are shown in Table 2. Catastrophizing at baseline was shown to be associated with more pain and disability at 6-month follow-up, but these associations were attenuated after adjustment for age, gender, baseline pain, disability, anxiety and depression. After adjustment for potential confounders, there was a positive and statistically significant association between ignoring sensation and both pain and disability at follow-up. Using self-statements and increasing behaviour were not statistically significantly associated with pain or disability at follow-up.

Table 3 present the associations between baseline coping strategies and pain and disability at 6-months follow-up, stratified by baseline pain or disability as appropriate. Catastrophizing, whether having below or above average pain or disability at baseline, was associated with more pain or disability at follow-up. However, once adjustments were made for confounders the association was no longer significant. In the higher baseline pain group, ignoring sensations was positively associated with pain at follow-up both in the crude (β =2.50, 95% CI 0.14, 4.86) and adjusted models (β =3.36, 95% CI 0.92, 5.80). This was not true for ignoring sensations in the lower pain group. On stratifying the sample into above and below median baseline disability, ignoring sensations, using self-statements and increasing behaviour were statistically non-significant.

## Discussion

This study investigated whether coping strategies were associated with higher levels of pain and disability (measured using the CPG) at six-month follow-up in older patients consulting primary care for musculoskeletal pain. Self-statements, ignoring sensations and increasing behaviour were not associated with pain related disability at six months. Similarly, self-statements and increasing behaviour were not predictive of pain at follow-up. However, ignoring sensations was related to increased pain at follow-up in those who presented with high baseline pain. Catastrophizing was predictive of increased pain and disability at follow-up but only in the unadjusted model, once confounders were taken into account catastrophizing was not significant.

Multiple studies have investigated active and passive coping styles in predicting pain and pain related disability in musculoskeletal disorders [21-23]. However, we are, to our knowledge, the first to present ignoring sensations as a separate entity. The initial analysis demonstrated the relationship between ignoring and more pain and disability at follow-up. Once stratified only the higher pain group showed this significant association. This suggests that the relationship between ignoring and pain is only maladaptive if your pain is initially severe. One previous study grouped ignoring pain sensations with “pain transformation” which also included coping styles such as pretending the pain is less severe. This paper showed “pain transformation” was related to more pain at follow-up in knee osteoarthritis [21]. Our study has shown those with higher baseline pain who ignore sensations do have higher pain at follow-up; further longitudinal research is needed in this area.

Pain catastrophizing is to focus on the magnitude and severity of pain sensations resulting in feeling pessimistic and helpless [11]. The relationship demonstrated between catastrophizing and pain and pain related disability, at first glance, is not consistent with other previously conducted prospective cohort studies investigating different musculoskeletal disorders [24,25]. In back pain patients, higher scores for catastrophizing were shown to be predictive of pain and pain related functional limitations at six-months (OR 2.3, 95% CI 1.1, 4.9) [24]. However this study did not adjust the results for age, gender, anxiety or depression. Catastrophizing has been identified to be a predictor of pain [25], disability [25] and depression [25,26] at follow-up. Also, both catastrophizing and depression have been showed to be predictive of disability [27]. These findings are of particular importance because they demonstrate catastrophizing can be predictive of depression but also depression is predictive of disability. Our crude results for catastrophizing do concur with previous research in the field but once appropriate adjustments are made, the relationship is no longer significant. It is hypothesed that the relationship between catastrophizing and pain and disability at follow-up is confounded by psychological factors such as depression. To our knowledge, there are no previous cohort studies that have adjusted for baseline depression and still shown that catastrophizing is predictive of pain and pain related disability in musculoskeletal disorders.

The present study has a relatively large sample size with satisfactory response rates extending to follow-up. This is a unique longitudinal study researching coping strategies in older adults within a primary care setting. Widely used validated tools were used to assess outcome and predictors. This study has accounted for anxiety and depression within the adjusted model which has strengthened the analysis and results. The PROG-RES study was not designed purely to examine the role of coping strategies in older people with musculoskeletal pain, hence the CSQ was modified to eight items excluding diverting attention, reinterpreting, and hoping and praying. It may have been beneficial to include self-efficacy [28] within the baseline questionnaire as this has previously been identified as a predictor of outcome in osteoarthritis [12]. A further limitation of this study is the lack of generalisability given the study was conducted in a geographical area where the ethnicity was mainly White British [29]. Additionally, it is possible that our stratified analyses are biased by regression to the mean. For example, in those people with higher levels of pain at baseline, it is likely that their level of pain will decrease over time regardless of any coping strategy that they might adopt. This means that our estimates of the effect of coping strategies may be biased towards the null. Similarly those with low levels of pain at baseline are likely to experience and increase in pain over time.

## Appendix 1

The amended version of the Coping Strategies Questionnaire

For each statement circle the number on the scale 0–6, that represents how you cope with your pain (where 0 is you never do that and 6 is you always do that)

1. It is terrible and I feel it is never going to go away (catastrophizing)

2. I don’t pay any attention to it (ignoring)

3. I tell myself I can’t let the pain stand in the way of what I have to do (using self-statements)

4. I do something active like household chores or projects (increasing behaviour)

5. I feel I can’t stand it anymore (catastrophizing)

6. I ignore it (ignoring)

7. I see it as a challenge and don’t let it bother me (self statements)

8. I do something I enjoy, such as watching TV or listening to music (ignoring)

## Conclusions

The findings of the present study suggest that ignoring in those with high baseline pain is a maladaptive coping strategy when used by older people with musculoskeletal pain. We have also demonstrated that catastrophizing is not predictive of pain or disability at follow-up once anxiety, depression, age and gender have been taken into account. Further research is needed to strengthen these findings as there are no comparable studies in the literature.

## Abbreviations

CSQ: The coping strategies questionnaire; CPG: The chronic pain grade; PROG-RES study: The prognostic research study.

## Competing interests

There are no conflicts of interest for any of the authors.

## Authors’ contributions

KB and SM drafted the manuscript, SM carried out the statistical analysis. All authors participated in the design of the study and read and approved the final manuscript.

## Pre-publication history

The pre-publication history for this paper can be accessed here:

http://www.biomedcentral.com/1471-2296/14/67/prepub
